# The Weak Microcavity as an Enabler for Bright and Fault-tolerant Light-emitting Electrochemical Cells

**DOI:** 10.1038/s41598-018-25287-x

**Published:** 2018-05-03

**Authors:** E. Mattias Lindh, Petter Lundberg, Thomas Lanz, Jonas Mindemark, Ludvig Edman

**Affiliations:** 1 The Organic Photonics and Electronics Group, Department of Physics, Umeå University, SE-90187 Umeå, Sweden; 2 Department of Chemistry - Ångström Laboratory, Uppsala University, SE-75121 Uppsala, Sweden

## Abstract

The light-emitting electrochemical cell (LEC) is functional at substantial active-layer thickness, and is as such heralded for being fit for low-cost and fault-tolerant solution-based fabrication. We report here that this statement should be moderated, and that in order to obtain a strong luminous output, it is fundamentally important to fabricate LEC devices with a designed thickness of the active layer. By systematic experimentation and simulation, we demonstrate that weak optical microcavity effects are prominent in a common LEC system, and that the luminance and efficiency, as well as the emission color and the angular intensity, vary in a periodic manner with the active-layer thickness. Importantly, we demonstrate that high-performance light-emission can be attained from LEC devices with a significant active-layer thickness of 300 nm, which implies that low-cost solution-processed LECs are indeed a realistic option, provided that the device structure has been appropriately designed from an optical perspective.

## Introduction

For a desired low-cost and upscaled production of large-area light-emitting and photovoltaic devices, a robust and comparatively thick active layer is an essential feature, since it renders the device fault-tolerant to defects and minimizes the risk of shorts through the active layer^1–3^. The light-emitting electrochemical cell (LEC) is an area-emissive device^4–7^ that can be fabricated with such cost-efficient^8^ and high-throughput solution-based techniques, e.g., slot-die and spray coating^9,10^, and inkjet and gravure printing^11–13^. Thereby it enables improved and new applications in a variety of fields, including healthcare^14^, illumination^15^ and signage^16,17^. A characteristic feature of the LEC technology is the *in-situ* formation of a p-n junction doping structure within the active material during operation^18–20^, which allows for low-voltage operation of LECs with thick active layers^4,21–23^.

However, although the electrical tolerance of LECs to the active-layer thickness has been well established through, e.g., the successful operation of mm-wide planar devices at a few volts drive voltage^22,23^, the corresponding optical dependence is less studied and comparatively poorly understood^24,25^. It has been demonstrated that losses due to doping-induced self-absorption become increasingly prominent as the active-layer thickness increases^26,27^, while an enhanced emission color or efficiency has been attributed to scattering^28,29^, microcavity^30,31^, and waveguide-coupling effects^32^.

Here, we report that the light-emission properties of a common LEC system are consistently periodic with the active-layer thickness, and, for instance, that the efficiency and luminance vary by one order of magnitude—while the forward emission color shifts from yellow, over orange and green, and back to yellow and orange—when the active-material thickness is increased from 100 to 380 nm. With the support of simulation results, we demonstrate that this periodic dependence is caused by weak microcavity effects. Our results provide important guidelines for how LECs with an active-layer thickness fit for fault-tolerant and upscaled solution fabrication should be designed for high-performance operation.

## Methods

### LEC fabrication

The fluorescent phenyl-substituted poly(para-phenylenevinylene) conjugated co-polymer (“Super Yellow”, trade name: PDY-132, Merck, Germany), the ion-dissolving and ion-transporting *n*-octyl carbonate-capped trimethylolpropane ethoxylate (TMPE-OC, synthesis described elsewhere)^33^, and the salt LiCF_3_SO_3_ (Aldrich), were separately dissolved in cyclohexanone (≥99.5%, Sigma-Aldrich) in a concentration of 10 mg/ml. These master solutions were mixed together in a mass ratio of Super Yellow:TMPE-OC:LiCF_3_SO_3_ = 1:0.2:0.03, and diluted with additional cyclohexanone to a final total solute concentration of 7 mg/ml. The resulting ink was spin-coated at different rotational speeds (500–1400 rpm, 1000 rpm/s, 60 s) onto pre-patterned and carefully cleaned indium tin-oxide (ITO) coated glass substrates (30 × 30 mm^2^, ITO thickness: 145 nm, Thin Film Devices, US) to form the active layer. The spin-coated active-layer film was dried at 70 °C for >6 h, and its thickness (d_AL_) was determined to range from 100–380 nm (mean of 4 measurements on each device) by contact profilometry (Bruker XT, US). A set of four Al top cathodes (thickness: 100 nm) was deposited onto the active layer by thermal evaporation under high vacuum (*p* < 5 · 10^−6^ mbar). The square emission area (A_LEC_) with side length w_LEC_ = 2 mm, was defined by the cathode–anode overlap. The emission area was protected from the ambient air by attaching a thin cover glass onto the cathode side of the device, using a UV-curable epoxy adhesive (Ossila, GBR). The edges of the glass substrates were blackened with a permanent marker pen to eliminate emission contributions from the substrate and wave-guided modes at large viewing angles. The material and device processing was executed in two interconnected N_2_-filled gloveboxes ([O_2_, H_2_O] <2 ppm).

### Optoelectronic characterization

**Figure 1:**
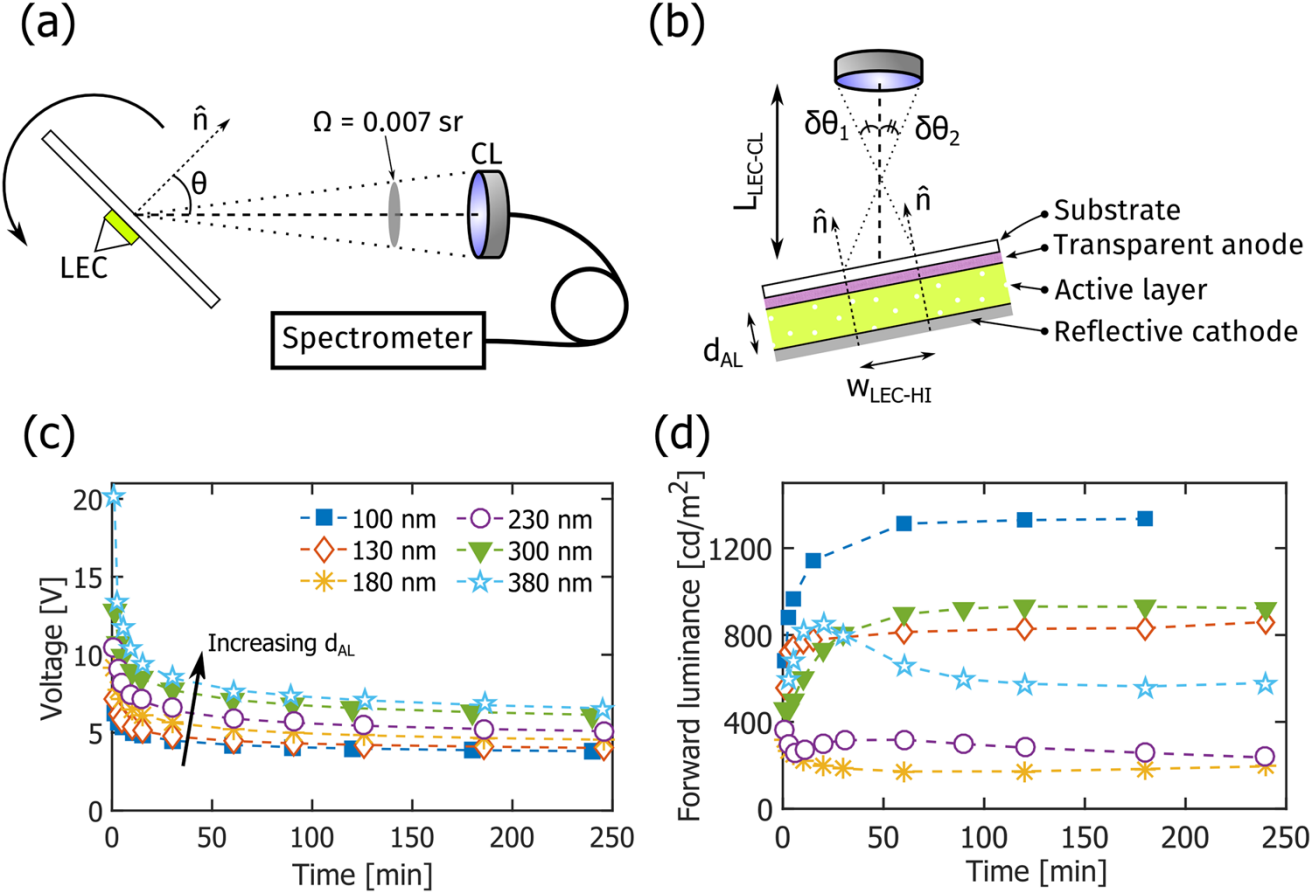
(**a**) A schematic of the custom-built automatic spectroscopic goniophotometry setup defining the viewing angle (θ) and the solid angle of collection (Ω). (**b**) A schematic of the LEC device structure and the positioning of the collimating lens (CL). The FWHM spread in the viewing angle, based on the half-intensity width of the LEC, w_LEC-HI_, is Δθ ≡ δθ_1_ + δθ_2_. Note that the dimensions are not drawn to scale. (**c**) The temporal evolution of the operating voltage for LEC devices with different active-layer thickness (d_AL_, as specified in the inset) during galvanostatic driving at a current density of 250 A/m^2^. (**d**) The corresponding temporal evolution of the forward luminance, as collected within a small solid angle of 0.007 sr.

The LEC devices were electrically driven and measured by a source-measure unit (2400, Keithley, US). All devices in this study were operated at a constant current density of 250 A/m^2^, with the voltage compliance set to 20 V. The angular dependence of the intensity and spectral shape of the resulting light emission was continuously (from turn-on to steady state) measured with a custom-built automated spectroscopic goniophotometer, as schematically depicted in Fig. 1(a,b). The LEC device was positioned in a non-interfering connection jig, and rotated around its polar axis with a stepper motor controlled by a LabVIEW virtual instrument (National Instruments, US). This rotation defined the viewing angle (θ), as identified in Fig. 1(a). A portion of the emitted light was collected by a collimating lens (CL, ∅_CL_ = 7.2 mm, F230 SMA-A, Thorlabs, Germany) positioned 75 mm (L_LEC-CL_) away from the LEC, which resulted in a small solid collection angle (Ω) of 0.007 sr. The non-zero extension of the LEC device resulted in a spread of the collected viewing angles, which for this particular device and measurement geometry was estimated to be Δθ = ±3.4°, assuming a homogeneous emission area and a half-intensity width of the device of w_LEC-HI_ = 1.6 mm; see Fig. 1(b). An optical fiber delivered the collected light to a CCD-array spectrometer (Flame-S, OceanOptics, US, linearity >99%, FWHM optical resolution <5 nm). The LabVIEW virtual instrument also administered the collection time and settings of the spectrometer, so that a large number of measurements could be carried out over a wide range of viewing angles (−80 < θ < 80°) within a short period of time (typically 35 different measurement angles within 1 min).

The spectral radiant intensity was derived from the raw spectrometer signal by multiplication with the setup’s response function for relative intensity. The luminous intensity was obtained by multiplication of the spectral radiant intensity with V_M_(λ) (the Judd-Vos modified CIE 2° Photopic luminosity curve)^34^ and subsequent spectral integration. As the size of the LEC device was much smaller than the distance between the LEC and the detector (w_LEC_ ≪ L_LEC-CL_), the luminance could be calculated by dividing the luminous intensity with the projected area of the LEC, i.e. A_LEC_ · cos(θ). The luminous power was calculated by integrating the measured luminous intensity over the forward hemisphere assuming azimuthal symmetry. The luminous efficacy was derived by dividing the luminous power with the driving current and voltage. All of the above optical data were measured in arbitrary units, and were either normalized or converted to absolute units using a reference value for the forward luminance, as measured with a luminance meter (Ω ≈ 3 · 10^−5^ sr, Konica-Minolta, LS-110, Japan). The relative standard error for the conversion was <2%.

The optical simulations were conducted with the emission module in SETFOS 4.4 (Fluxim AG, Switzerland), using values for the refractive index and the photoluminescence spectrum of the pristine constituent materials, as provided by the module^35^. The active layer was considered transparent and the emitting dipoles were represented by a delta function positioned at a distance of 0.4 · d_AL_ away from the reflective cathode. The layers had translational symmetry in the device plane and were thus solely represented in the normal direction. The presented simulation results correspond to unpolarized emission in order to be comparable to the experimental data.

### Data availability

All relevant data are available from the authors upon request.

## Results and Discussion

The custom-built automatic spectroscopic goniophotometer, used for the angular and spectral characterization of the light emission from the LECs, is schematically presented in Fig. 1(a,b). The LEC device under study was rotated around its polar axis, with the azimuthal angle and the location of the collimating lens (CL) fixed; the viewing angle (θ) was measured with respect to the LEC substrate normal ($\hat{{\rm{n}}}$). The relatively small size of the CL and the LEC device, in combination with the long distance between them, resulted in a desired small solid collection angle (Ω) of 0.007 sr, and a FWHM viewing-angle spread (Δθ) of ±3.5° (more details on the experimental setup are available in the Methods section).

Figure 1(b) also depicts the LEC device structure. The active layer was sandwiched between a reflective Al cathode and a transparent (and weakly reflective) ITO anode, with the latter being positioned on a transparent (and weakly reflective) glass substrate. The active layer consisted of the often employed fluorescent conjugated polymer Super Yellow blended with a (TMPE-OC + LiCF_3_SO_3_) electrolyte developed for high-performance LEC operation^33^. The influence of the active-layer thickness (d_AL_) on the light-emission characteristics was a prioritized topic in this study, and to facilitate the below discussion we have categorized the different LECs as follows: “thin” devices with d_AL_ = 100, 130 nm, “intermediate” devices with d_AL_ = 180, 230 nm, and “thick” devices with d_AL_ = 300, 380 nm. In total, we have fabricated and characterized more than 25 independent devices, and we found that the performance variation between devices of the same thickness was minor.

Figure 1(c,d) display the temporal evolution of the voltage and forward luminance, respectively, for the LEC devices with different active-layer thickness when driven by a constant current density of 250 A/m^2^, whereas key steady-state performance data are summarized in Table 1. The voltage is observed to decrease in a continuous manner for all devices during the initial turn-on process. This behavior is in agreement with the characteristic LEC operation, where the ions first redistribute towards the electrode interfaces to form electric double layers that assist with the electronic charge injection, and where the remaining ions thereafter assist with the conductivity-enhancing electrochemical doping of the electroluminescent compound (here Super Yellow), so that a p-n junction doping structure forms^4,36,37^. The observation that the voltage increases slightly with increasing active-layer thickness can be explained by that the electric resistance of a thicker material is higher than that of its thinner identical counterpart, but it is notable (and expected) that the steady-state operating voltage only increased by a factor of 1.7 when the active-layer thickness increased from 100 to 380 nm.

**Table 1:** Steady-state optoelectronic performance data of LECs with different active-layer thicknesses.

Active-layer thickness, d_AL_ [nm]	Current density [A/m^2^]	Operating voltage [V]	Forward luminance [cd/m^2^]	Forward current efficacy [cd/A]	Luminous efficacy [lm/W]	Chromaticity coordinates (x, y)	Lambertian correction factor, K_L_
100	250	3.87	1335	5.34	4.40	(0.51, 0.49)	1.02
130	250	4.10	832	3.33	2.95	(0.52, 0.47)	1.16
180	250	4.65	186	0.74	0.91	(0.58, 0.42)	1.82
230	250	5.23	260	1.04	0.43	(0.37, 0.62)	0.68
300	250	6.32	941	3.76	1.39	(0.47, 0.52)	0.74
380	250	6.77	570	2.28	1.42	(0.57, 0.42)	1.34

The Lambertian correction factor, K_L_, is defined in Equation (1).

The measured data for the forward luminance are, however, more difficult to explain. All devices turn on and emit light at a significant luminance (>100 cd/m^2^) within 3 s, but both the qualitative temporal behavior and the peak luminance exhibit a complicated dependence on the active-layer thickness, as presented in Fig. 1(d) and Table 1. From a sole charge-balance point-of-view^38^, the expected behavior is that the luminance will increase with time, since electric double layer and p-n junction formation will result in a more balanced hole/electron injection and a concomitant higher exciton formation rate. And from a sole self-absorption perspective^26^, the thinner devices should feature a higher peak luminance, as fewer photons should be lost to absorption during the passage through a thinner active layer.

For the thin devices, we do observe that the luminance increases monotonically with time, and that the peak luminance is higher for the thinner 100 nm LEC, at 1335 cd/m^2^, than for the 130 nm LEC. In contrast, the luminance from the two intermediate devices initially *decreases* with time, and the peak luminance is higher for the thicker 230 nm LEC than for the 180 nm LEC. Similar to the thin devices, the luminance from the two thick devices initially increases with time, and the peak luminance from the thinner 300 nm LEC is higher, at 940 cd/m^2^, than the luminance from the 380 nm LEC. Moreover, in addition to this non-systematic variation of the forward luminance with active-layer thickness, we also call attention to the unexpected observation that the peak luminance from the 300 nm LEC is higher than all but the thinnest 100 nm LEC. Furthermore, we note that the slope of the forward luminance for the 230 and 380 nm thick LECs changes sign after ~30 min of operation, but that no indication of degradation can be gleaned from the corresponding voltage evolution graph (Fig. 1c).

It is accordingly clear that simple charge-balance^38^ and self-absorption^26^ arguments cannot fully explain the observed forward luminance dependency on active-layer thickness. Thus, to better understand the temporal evolution of the optoelectronic properties of operating LECs, we have systematically collected angularly and spectrally resolved intensity data. In order to facilitate the presentation of these data, we define, and from here on employ, three key stages of operation: the “initial” stage at 1–2 min, the “transition” stage between 5 and 60 min, and the “steady-state” stage at ~180 min.

**Figure 2:**
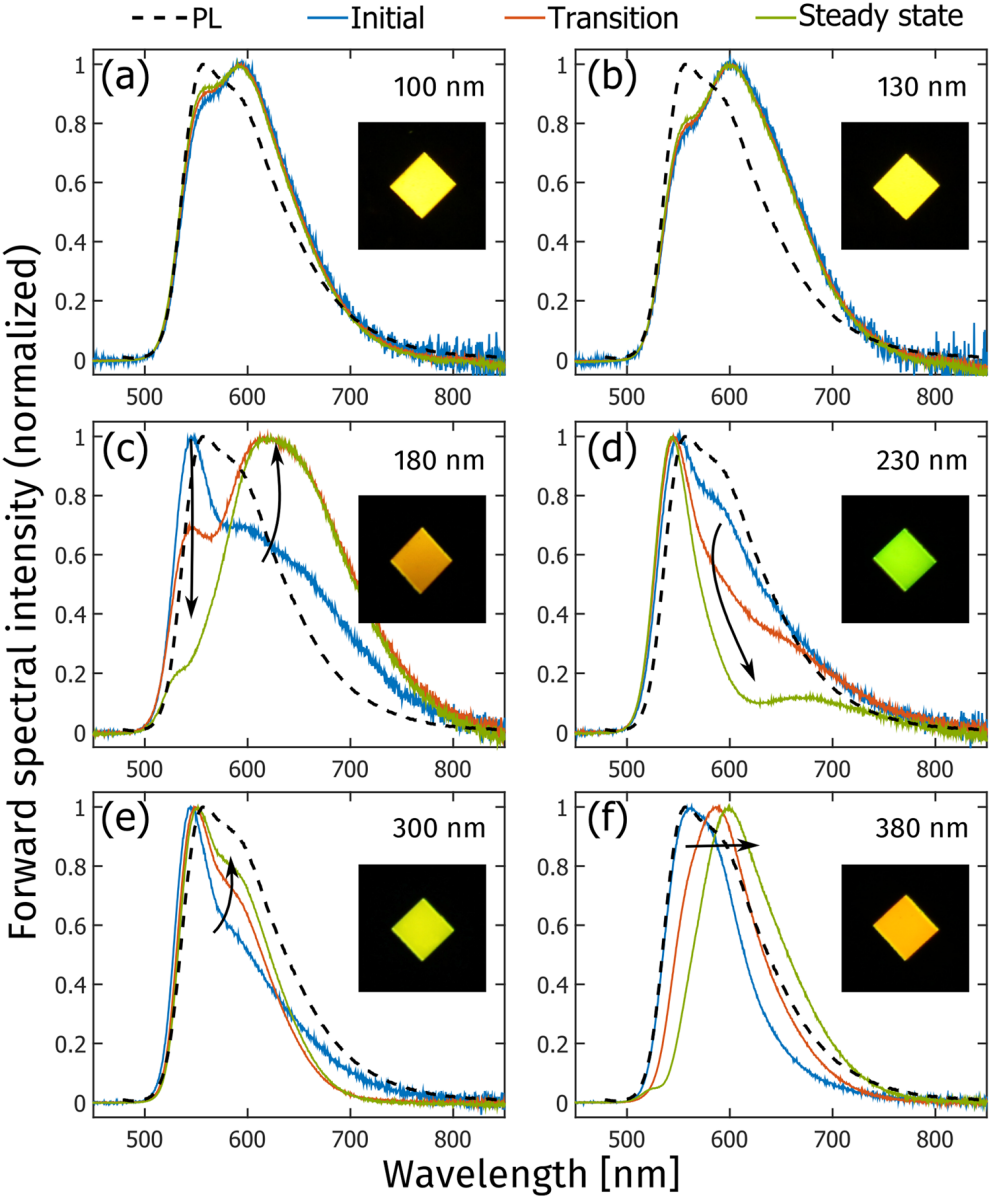
The temporal evolution of the normalized forward electroluminescence spectrum of LECs with different active-layer thickness, as identified in the upper right corner. The arrows and colors indicate increasing time from 1–2 min (initial, blue line), over 5–60 min (transition, red line), to 180 min (steady-state, green line). The photoluminescence spectrum of a thin film of the conjugated-polymer emitter, Super Yellow, is included as the black dashed line for reference. The inset photographs show the corresponding forward emission color during steady-state operation.

Figure 2 presents the temporal evolution of the normalized forward electroluminescence (EL) spectrum of LEC devices with different active-layer thickness, and photographs of the steady-state emission. The steady-state xy-chromaticity coordinates (in the CIE 1931 color space) in the forward direction are presented in Table 1, while the temporal evolution of the chromaticity coordinates is displayed in Fig. S1 in the Supporting Information. The EL spectra of the thin devices in Fig. 2(a,b) are essentially invariant with time and also highly reminiscent of the PL spectrum of Super Yellow (xy = [0.49, 0.50]), with the minor difference being that the second vibronic peak at ~600 nm is stronger in the EL. Consequently, the steady-state forward emission color from the thin devices, as displayed in the inset, is the characteristic yellow of Super Yellow.

The contrast with the evolution of the EL spectra for the intermediate devices in Fig. 2(c,d) is striking. Both initial EL spectra are similar to the PL spectrum of Super Yellow, but with the second vibronic peak now being suppressed. The subsequent temporal evolution is dramatic; for the 180 nm LEC the 550 nm main peak gradually disappears, while a new broad and distinctly red-shifted peak centered at ~620 nm emerges. This significant change in the EL spectrum is manifested in the orange emission color (xy = [0.58, 0.42]) in the corresponding inset photograph. The temporal change for the 230 nm LEC is also significant, but here the EL spectrum is blue-shifted instead of red-shifted. More specifically, the second vibronic peak at ~600 nm essentially disappears with increasing time, so that the entire emission envelope narrows, and the forward emission color of the LEC turns yellow-green (xy = [0.37, 0.62]) as displayed in the inset photograph.

In comparison to the intermediate devices, the temporal changes in the forward EL spectrum for the thick devices in Fig. 2(e,f) are less dramatic, albeit still significant. The 300 nm LEC features a narrow initial EL spectrum, with a suppressed second vibronic peak in comparison to the PL spectrum of Super Yellow. With time, the second vibronic peak grows in amplitude and the initial red tail disappears, resulting in a greenish yellow emission color (xy = [0.42, 0.57]). For the 380 nm LEC device, the entire EL spectral envelope is red-shifted with time, as manifested in an EL peak shift from 560 to 600 nm, and a yellowish orange emission color in the forward direction (xy = [0.57, 0.42]).

It is interesting that the intermediate devices feature the lowest steady-state forward luminance (Fig. 1d), the most pronounced EL-spectrum evolution, and the largest spectral deviation from the PL of the Super Yellow emitter. However, both the luminance and the EL-spectrum were measured in the forward direction within a small solid collection angle, and in order to investigate whether the same trend holds true for other viewing angles, and for the integrated emission performance, we now turn our attention to angularly resolved measurements.

**Figure 3:**
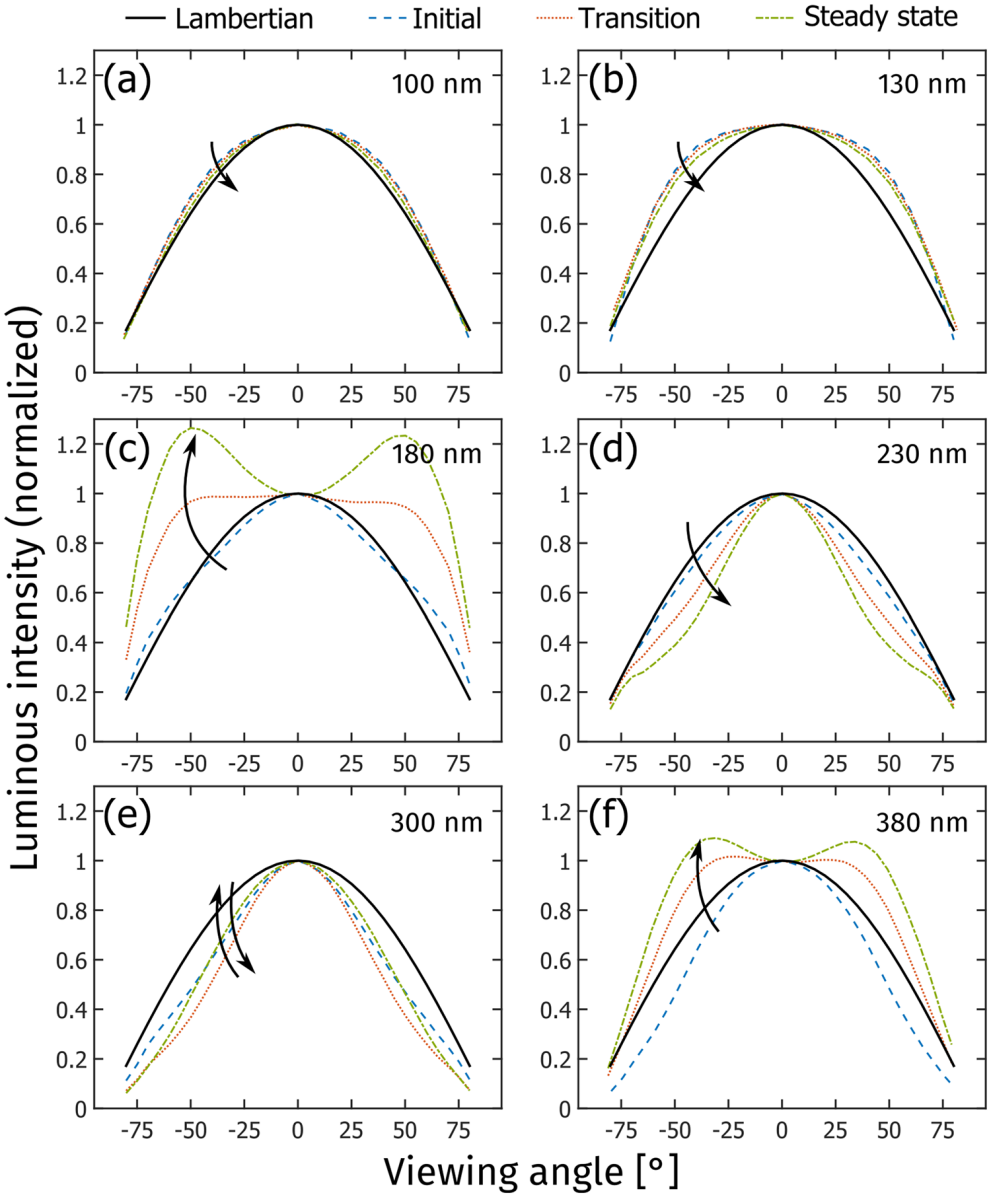
(**a**) The temporal evolution of the luminous efficacy for LECs with different active-layer thickness: 100 nm (solid blue squares), 130 nm (open red diamonds), 180 nm (yellow stars), 230 nm (open purple circles), 300 nm (solid green triangles), and 380 nm (open light-blue stars). (**b**) The steady-state luminous efficacy (solid purple pentagons) and the steady-state forward luminance (yellow crosses) as a function of the active-layer thickness. (**c**) The steady-state Lambertian correction factor (K_L_) as a function of active-layer thickness. The grey dotted line (K_L_ = 1) is the result of a Lambertian emitter. (**d**) The steady-state emission peak wavelength in the forward direction (solid squares), and at a viewing angle of 70° (open circles), as a function of the active-layer thickness. (**e**) The simulated K_L_ as a function of active-layer thickness. (**f**) The simulated emission peak wavelength in the forward direction (solid blue squares), and at a viewing angle of 70° (open red circles), as a function of the active-layer thickness.

Figure 3 presents the temporal evolution of the normalized luminous intensity as a function of viewing angle, i.e. the luminous intensity distribution, for LECs with different active-layer thickness. Surface-emitting devices, such as LECs and organic light-emitting diodes, can—but need not—feature a Lambertian luminous intensity distribution. A Lambertian emitter is defined by that the luminance is invariant with viewing angle, which in our measurement setup is only true if the luminous intensity is directly proportional to the cosine of the viewing angle^39^. The luminous intensity distribution for a Lambertian emitter is included as the solid black line in Fig. 3.

Figure 3(a,b) show that the thin LECs feature close-to-Lambertian luminous intensity distributions that are essentially invariant with time. This observation is in agreement with previous findings for similarly thin conjugated-polymer based LECs^28^. The intermediate LECs in Fig. 3(c,d) also display a Lambertian luminous intensity distribution during the initial operation, but the temporal evolution is drastic. For the 180 nm LEC, a strong angular emission centered at θ ≈ ±45° is observed to grow with time, and during steady-state operation it is almost twice as intense as that of a Lambertian emitter. In contrast, the angular emission of the 230 nm LEC is decreasing with time, and the steady-state intensity at θ ≈ ±45° is close to half of that of the Lambertian reference. The luminous intensity distribution of the thick LECs is presented in Fig. 3(e,f), and both devices display an initial angular emission that is weaker than that of a Lambertian emitter. While the luminous intensity distribution of the 300 nm LEC only shows minor temporal evolution, the angular emission from the 380 nm LEC increases markedly so that the steady-state angular emission lobes, centered at θ ≈ ±30°, are about 30% more intense than the Lambertian reference.

The data in Fig. 3 do not only provide important information on the direction in which light is emitted, but also serve as a reminder to why an often employed simplification procedure in the field should be exercised with caution. One of the most important metrics for a light-emitting device is the luminous efficacy (or power conversion efficiency), which informs on the efficiency with which the input electric power is converted into visible illumination power. A common method for calculating the luminous efficacy is to measure the luminance in the forward direction (L_θ=0_), multiply this value with π and the emitting area, and divide this Lambertian-derived luminous power with the input electric power. However, this procedure is solely valid if the device emits with a Lambertian luminous intensity distribution, which is demonstrated to hold true only under some specific conditions for the herein investigated LECs (see Fig. 3). A measure on the relative error that result from employing the Lambertian assumption is provided by the “Lambertian correction factor”, K_L_, which is the quotient between the total emitted luminous power, Φ_tot_, and the Lambertian-derived luminous power

1 $${{\rm{K}}}_{{\rm{L}}}=\frac{{{\rm{\Phi }}}_{{\rm{tot}}}}{{\rm{\pi }}\cdot {{\rm{L}}}_{{\rm{\theta }}=0}\cdot {{\rm{A}}}_{{\rm{LEC}}}},$$

where Φ_tot_ was measured by integration of the luminous intensity distribution over the forward hemisphere^40^. Table 1 presents the steady-state values for K_L_ at different active-layer thickness, and it is notable that while the Lambertian assumption is a good approximation for the thinnest 100 nm LEC (K_L_ = 1.02), it results in significant errors for the other LEC devices, as exemplified by K_L_ = 1.82 for the 180 nm LEC and K_L_ = 0.68 for the 230 nm LEC. Thus, for the 180 nm LEC, the employment of the Lambertian assumption would result in an *underestimation* of the true luminous efficacy by 45%, while the same assumption for the 230 nm LEC *overestimates* the luminous efficacy by 47%. For this reason, all values for the luminous efficacy in this report were derived using the measured total luminous power (see Methods section for details).

**Figure 4:**
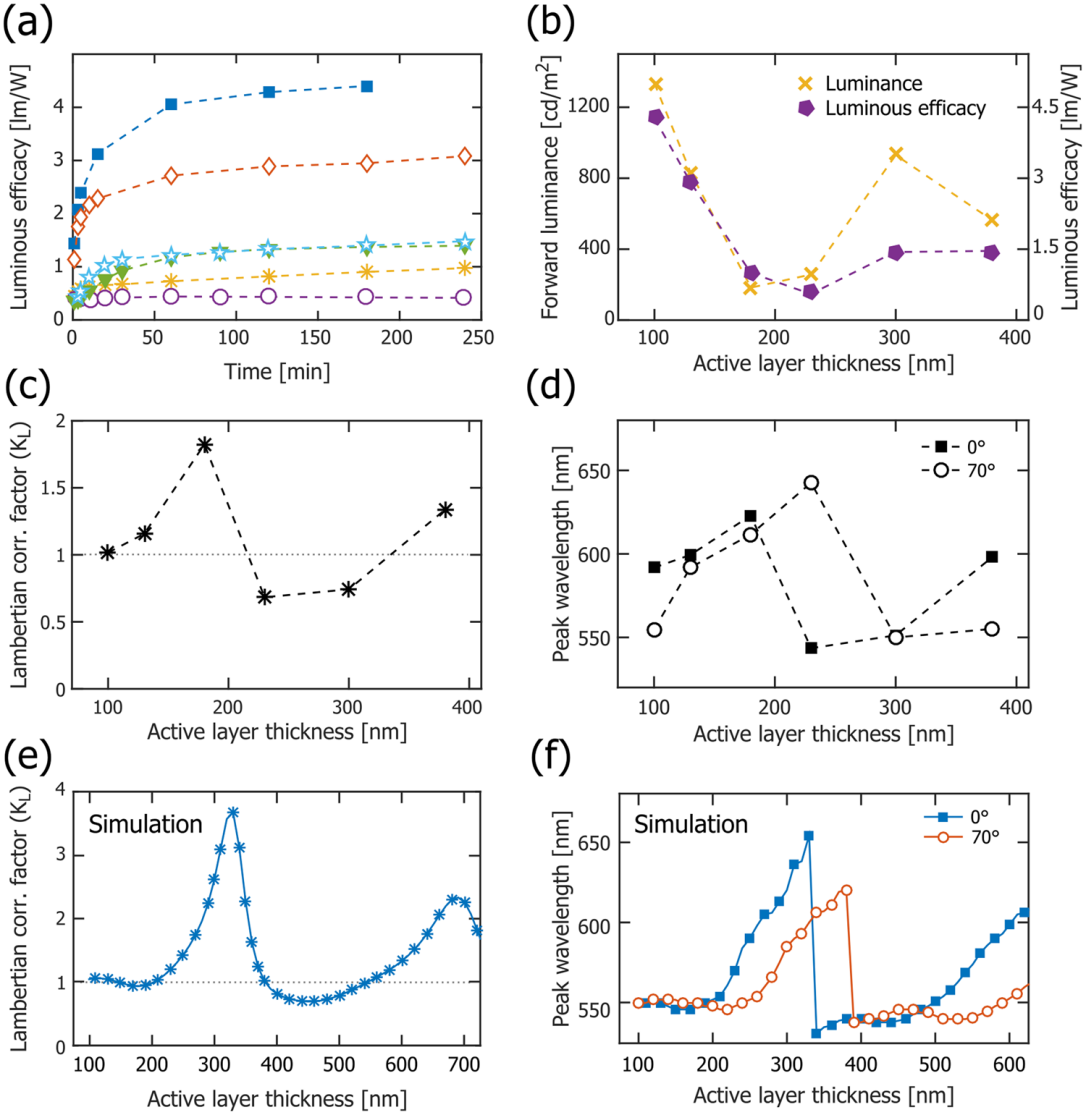
The temporal evolution of the luminous intensity as a function of viewing angle for LECs with different active-layer thickness, as identified in the upper right corner. The colors and arrows indicate increasing time from 1–2 min (initial, dashed blue line), over 5–60 min (transition, dotted red line), to 180 min (steady-state, dash-dotted green line). The black solid line is included as a Lambertian reference.

Figure 4(a) displays the temporal evolution of the (appropriately measured) luminous efficacy for LEC devices with different active-layer thickness when driven by a current density of 250 A/m^2^. The luminous efficacy is observed to increase monotonously with time for all active-layer thicknesses, which is in agreement with the LEC-characteristic *in-situ* formation of electric-double layers and a p-n junction doping structure, since these processes will result in a balanced electron and hole injection and a lowered drive voltage. Less intuitively, Fig. 4(b) reveals that the steady-state luminous efficacy (solid purple pentagons) has an undulating dependence on the active-layer thickness, with a local minimum for the 230 nm LEC. We note with interest that a similar periodic behavior is displayed also for the forward luminance (Fig. 4b, yellow crosses), the Lambertian correction factor (Fig. 4c), and the emission peak wavelength at different viewing angles (Fig. 4d).

The consistent periodic dependence on the active-layer thickness suggests that optical microcavity effects, i.e. the existence of coherent standing waves between (strongly and weakly) reflective interfaces in the device structure, could be a plausible origin^41–44^. In our particular device geometry, the strongly reflective interface is the active-layer/aluminum-cathode interface, while the weakly reflective interfaces are the active-layer/ITO and the ITO/glass interfaces. To investigate whether it is possible to theoretically replicate this experimentally observed behavior, we have performed a coherent optical simulation study. In order to minimize the number of “free” parameters in the simulation, we focused on the steady-state behavior and made the following simplifications: the active layer is transparent and free from electrolyte, the refractive indices of the constituent materials are the tabulated pristine values provided by the simulation software, and the spatial distribution of the emitting dipoles is described by a delta function positioned at a distance of 0.4 · d_AL_ from the reflective cathodic interface (see Fig. 1b). This emitter position was allowed to vary in the simulation, and the selected value provided the best agreement with the experimental data.

Figure 4(e,f) present the simulated wavelength dependence for the Lambertian correction factor and the two emission peak wavelengths, respectively, and the qualitative agreement with the corresponding experimental data in Fig. 4(c,d) must be considered good: the undulating Lambertian correction factor is well matched, and both the shape of the two emission peak wavelength curves and the “phase shift” between the forward (θ = 0°) and the θ = 70° emission are captured by the simulation. The fact that the quantitative values for the wavelength and the correction factor are off in the simulation is attributed to the selected simple model, and we anticipate that a better match would be attained with more realistic values for the various material parameters. Nevertheless, the consistent periodic active-layer thickness dependence of important performance metrics, and the ability of a coherent simulation to qualitatively replicate this behavior, implies that optical microcavity effects must be considered in a comprehensive analysis of LEC performance. Although the above discussion was limited to device data recorded at steady-state, we emphasize that microcavity effects most probably are in play also during the dynamic turn-on process depicted in Figs 1–3. However, it should be noted that a corresponding temporal simulation would be more cumbersome to perform, owing to the evolution of the doping structure, with concomitant temporal changes in the refractive indices of the constituent materials and the position of the excitons^24,31,45,46^.

We have in this study focused on the emission from a Super Yellow-based LEC, but the results should be applicable also for LECs based on other emitters. The emission from an optical microcavity is the product of the intrinsic emission spectrum of the emitter and the cavity gain, which implies that broad emitters, such as conjugated polymers^4,33,45,47,48^ and ionic transition metal complexes^49–53^ (the two most common emitter materials in LECs), can encompass multiple cavity modes depending on the active-layer thickness and the viewing angle; this feature is clearly displayed in Fig. 2 ^54,55^. For the attainment of white light emission^56^ this issue is compounded as the emission color could change with viewing angle, whereas the efficiency of narrow emitters can be boosted by an appropriate design of the optical cavity^57^. It is further to be noted that the introduction of scattering layers^58^, strongly absorbing layers^59^, or the employment of rough surfaces^58,60^, can inhibit the coherence and as a consequence attenuate the microcavity effects^61^.

We finally want to reinforce an important take-home message of this study: although the electrical properties of a functional LEC device are notably independent on the thickness of the active material (see Fig. 1c), this is not the case for the optical properties. More specifically, while an increase in the active-layer thickness from 100 to 380 nm resulted in an increase in the steady-state drive voltage by merely a factor of 1.7, the forward luminance and the luminous efficacy varied (in a periodic manner) by one order of magnitude over the same thickness interval (see Table 1). This strong dependence on the active-layer thickness has implications for the appropriate evaluation of new materials in LEC devices, since an incorrectly selected active-layer thickness will result in a sub-par device performance that is due to a poorly designed optical cavity and not to a non-performing material. Most importantly, since a reasonably thick active layer is a prerequisite for fault-tolerant and low-cost solution-based fabrication, it is comforting that good luminous (and electrical) performance could be attained from the LEC device with a 300 nm thick active layer.

## Conclusions

Through systematic experimentation and simulation, we demonstrate that optical microcavity effects can significantly influence the luminous performance of LEC devices, and that the optical output as a consequence displays a periodic dependency on the active-layer thickness. Specifically, the peak luminance and efficiency from a Super Yellow-based LEC are observed to vary by one order of magnitude, and the forward emission color change—from yellow, over orange and green, and back to yellow and orange—when the active-material thickness is increased from 100 to 380 nm. We further report that the light intensity distribution exhibits a complex dependence on both time and active-layer thickness, and that the commonly employed assumption of Lambertian emission is inappropriate for all but the thinnest 100 nm LEC. Finally, our results yield support for that high-performance LECs can be fabricated with low-cost, solution-based fabrication methods, but that the attainment of good luminous performance from such devices is directly dependent on an appropriate design of the optical microcavity.

## Electronic supplementary material

Supplementary Information
