# Publisher Correction: The Weak Microcavity as an Enabler for Bright and Fault-tolerant Light-emitting Electrochemical Cells

**DOI:** 10.1038/s41598-018-26760-3

**Published:** 2018-06-01

**Authors:** E. Mattias Lindh, Petter Lundberg, Thomas Lanz, Jonas Mindemark, Ludvig Edman

**Affiliations:** 1 The Organic Photonics and Electronics Group, Department of Physics, Umeå University, SE-90187 Umeå, Sweden; 2 Department of Chemistry - Ångström Laboratory, Uppsala University, SE-75121 Uppsala, Sweden

Correction to: *Scientific Reports* 10.1038/s41598-018-25287-x, published online 03 May 2018

This Article contains an error in the Figure legends of Figure 3 and Figure 4. The legends of these Figures were inadvertently switched.

The legend of Figure 3:

“**(a)** The temporal evolution of the luminous efficacy for LECs with different active-layer thickness: 100 nm (solid blue squares), 130 nm (open red diamonds), 180 nm (yellow stars), 230 nm (open purple circles), 300 nm (solid green triangles), and 380 nm (open light-blue stars). **(b)** The steady-state luminous efficacy (solid purple pentagons) and the steady-state forward luminance (yellow crosses) as a function of the active-layer thickness. **(c)** The steady-state Lambertian correction factor (K_L_) as a function of active-layer thickness. The grey dotted line (K_L_ = 1) is the result of a Lambertian emitter. **(d)** The steady-state emission peak wavelength in the forward direction (solid squares), and at a viewing angle of 70° (open circles), as a function of the active-layer thickness. **(e)** The simulated K_L_ as a function of active-layer thickness. **(f)** The simulated emission peak wavelength in the forward direction (solid blue squares), and at a viewing angle of 70° (open red circles), as a function of the active-layer thickness.”

should read:

“The temporal evolution of the luminous intensity as a function of viewing angle for LECs with different active-layer thickness, as identified in the upper right corner. The colors and arrows indicate increasing time from 1–2 min (initial, dashed blue line), over 5–60 min (transition, dotted red line), to 180 min (steady-state, dash-dotted green line). The black solid line is included as a Lambertian reference.”

The legend of Figure 4:

“The temporal evolution of the luminous intensity as a function of viewing angle for LECs with different active-layer thickness, as identified in the upper right corner. The colors and arrows indicate increasing time from 1–2 min (initial, dashed blue line), over 5–60 min (transition, dotted red line), to 180 min (steady-state, dash-dotted green line). The black solid line is included as a Lambertian reference.”

should read:

“**(a)** The temporal evolution of the luminous efficacy for LECs with different active-layer thickness: 100 nm (solid blue squares), 130 nm (open red diamonds), 180 nm (yellow stars), 230 nm (open purple circles), 300 nm (solid green triangles), and 380 nm (open light-blue stars). **(b)** The steady-state luminous efficacy (solid purple pentagons) and the steady-state forward luminance (yellow crosses) as a function of the active-layer thickness. **(c)** The steady-state Lambertian correction factor (K_L_) as a function of active-layer thickness. The grey dotted line (K_L_ = 1) is the result of a Lambertian emitter. **(d)** The steady-state emission peak wavelength in the forward direction (solid squares), and at a viewing angle of 70° (open circles), as a function of the active-layer thickness. **(e)** The simulated K_L_ as a function of active-layer thickness. **(f)** The simulated emission peak wavelength in the forward direction (solid blue squares), and at a viewing angle of 70° (open red circles), as a function of the active-layer thickness.”

