# Supplementary material for: The Weak Microcavity as an Enabler for Bright and Fault-tolerant Light-emitting Electrochemical Cells
Source: Sci Rep. 2018 May 3;8:6970. doi: 10.1038/s41598-018-25287-x (PMC5934366; doi:10.1038/s41598-018-25287-x)
Supplement: Supplementary file 1 — Supplementary Information [file 41598_2018_25287_MOESM1_ESM.docx]

**Supplementary Information for**

**The Weak Microcavity as an Enabler for Bright and Fault-tolerant Light-emitting Electrochemical Cells**

E. Mattias Lindh^1^, Petter Lundberg^1^, Thomas Lanz^1^, Jonas Mindemark^1,2^ and Ludvig Edman^1^*

^1^ The Organic Photonics and Electronics Group, Department of Physics, Umeå University, SE-90187 Umeå, Sweden
^2^ Department of Chemistry - Ångström Laboratory, Uppsala University, SE-75121 Uppsala, Sweden
*Corresponding author e-mail address: ludvig.edman@umu.se

**The temporal evolution of the forward chromaticity coordinates**

Fig. S1 presents the temporal evolution of the xy-chromaticity coordinates (in the CIE 1931 color space) of the forward electroluminescence from LECs with different active-layer thickness (as specified in the legend). The PL of a thin film of Super Yellow is also indicated by the large yellow star for reference. All LEC devices exhibit emission that is located along the red-green border of the color space. The thin devices (100 & 130 nm) feature a minor evolution of the chromaticity coordinates, whereas the intermediate (180 & 230 nm) and thick (300 & 380 nm) devices display distinct color shifts.


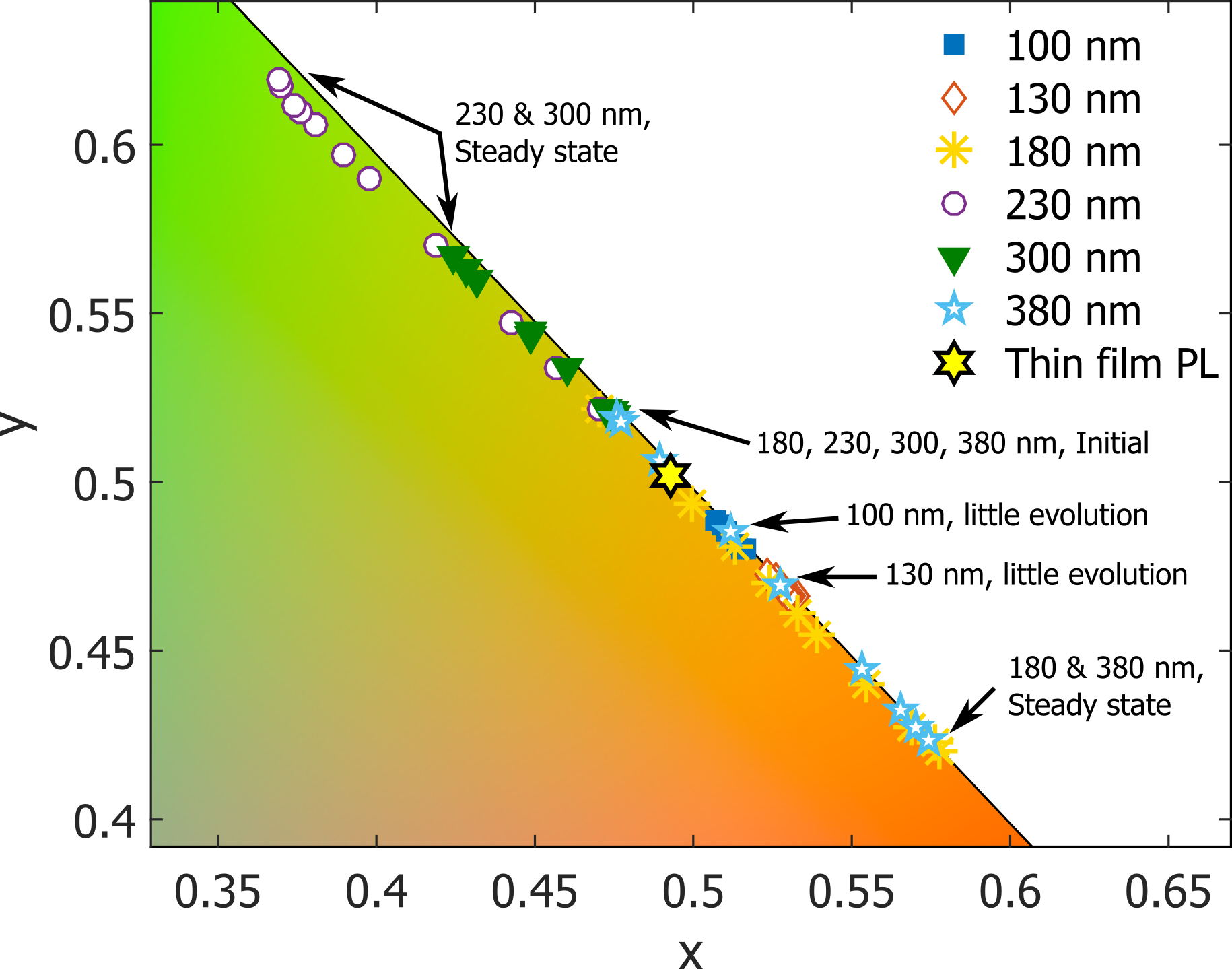


Figure S1. The temporal evolution of the CIE 1931 xy-chromaticity coordinates of the forward electroluminescence at different active-layer thicknesses (as specified in the legend).
